# At Long Last: The Me_3_Si Group as a Masked Alcohol

**DOI:** 10.1002/anie.202017157

**Published:** 2021-01-26

**Authors:** Avijit Roy, Martin Oestreich

**Affiliations:** ^1^ Institut für Chemie Technische Universität Berlin Strasse des 17. Juni 115 10623 Berlin Germany

**Keywords:** alcohols, density functional calculations, hypervalent compounds, silicon, Tamao–Fleming oxidation

## Abstract

For a long time, the Me_3_Si group has been ostracized from the family of aryl‐ and heteroatom‐substituted congeners for the difficulties associated with its further chemoselective manipulation into another synthetically useful functional group. A hypervalent iodine reagent has now been shown to do exactly that by electrophilic demethylation. Coupled with the Tamao–Fleming oxidation, the Me_3_Si group becomes a placeholder for a hydroxy group.

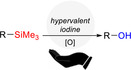

Silicon, carbon's closest neighbor in the periodic table, forms strong covalent bonds with carbon. Due to the more electropositive nature of silicon, carbon−silicon bonds are however more polarized and, hence, more vulnerable to nucleo‐ and electrophilic attack, respectively, as compared to carbon−carbon bonds. Such fragility of carbon−silicon bonds had been realized a century ago for the first time by the pioneer of silicon chemistry, Frederick S. Kipping.^[1]^ His serendipitous discovery of the acid‐mediated cleavage of C(sp^2^)−Si bonds in arylsilanes (=protodesilylation) was further advanced by Colin Eaborn,^[2]^ and eventually streamlined by Ian Fleming for the synthesis of alcohols from arylsilanes^[3]^ [Eq. 1]. The overall procedure is the now called Tamao–Fleming oxidation (gray box).^[4]^ Unactivated tetraalkylsilanes were found to be too unreactive though, and the oxidative degradation of trialkylsilyl groups, especially the Me_3_Si group, as masked hydroxy groups is still elusive.^[5]^

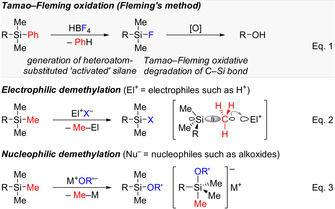



Following a similar strategy, several attempts have been made for the demethylation of trimethylsilane derivatives employing strong Brønsted acids [Eq. 2]. In 1953, Sommer and co‐workers reported a concentrated sulfuric acid‐aided synthesis of aliphatic organosiloxanes through the cleavage of one methyl group from the trimethylsilyl moiety.^[6a]^ Kinetic studies confirmed a pseudo‐first order character of the reaction, involving an “electrophilic attack” on the carbon center followed by the formation of methane and a (at least formally) silylium‐ion intermediate in the rate‐determining step.^[6b]^ Later, a series of experiments was described by O'Brien on the stepwise protolytic cleavage of C(sp^3^)−Si bonds in tetra‐ and trialkylsilanes employing acid systems stronger than concentrated sulfuric acid, including HSO_3_F/SbF_5_/SO_2_, HSO_3_F/SO_2_, and HSO_3_F.^[7]^ The downside of these approaches is the difficulty of isolating the products from super acidic media. Independent reports by Olah^[8]^ and Demuth^[9]^ about the electrophilic demethylation overcame this problem. By using triflic acid, one of the methyl groups in tetramethylsilane is selectively protonated, affording trimethylsilyl triflate in almost quantitative yield. Our laboratory recently showed that Reed's carborane acids are also capable of performing selective protonation of one alkyl group, thereby providing access to various counteranion‐stabilized silylium ions.^[10]^ As to nucleophilic demethylation, hard oxygen nucleophiles have been mostly employed^[11]^ but, unless intramolecular, modest substrate specificity has so far limited synthetic applications [Eq. 3].

By clever combination of those electro‐ and nucleophilic activation strategies, Matsunaga, Yoshino, and co‐workers accomplished the chemoselective C(sp^3^)−Si bond cleavage in tetraalkylsilanes using iodine tris(trifluoroacetate) (ITT) and merged their new method with the aforementioned oxidative degradation of the heteroatom‐substituted silicon intermediate [Eq. 4].^[12]^ That two‐step sequence is reminiscent of the original report on the Fleming oxidation.^[3a]^ The use of an electrophilic iodine reagent is a judicious choice for such transformation as evidenced by an early work from Eaborn^[13]^ [Eq. 5] and an unexpected finding by Shindo and co‐workers^[14]^ [Eq. 6].
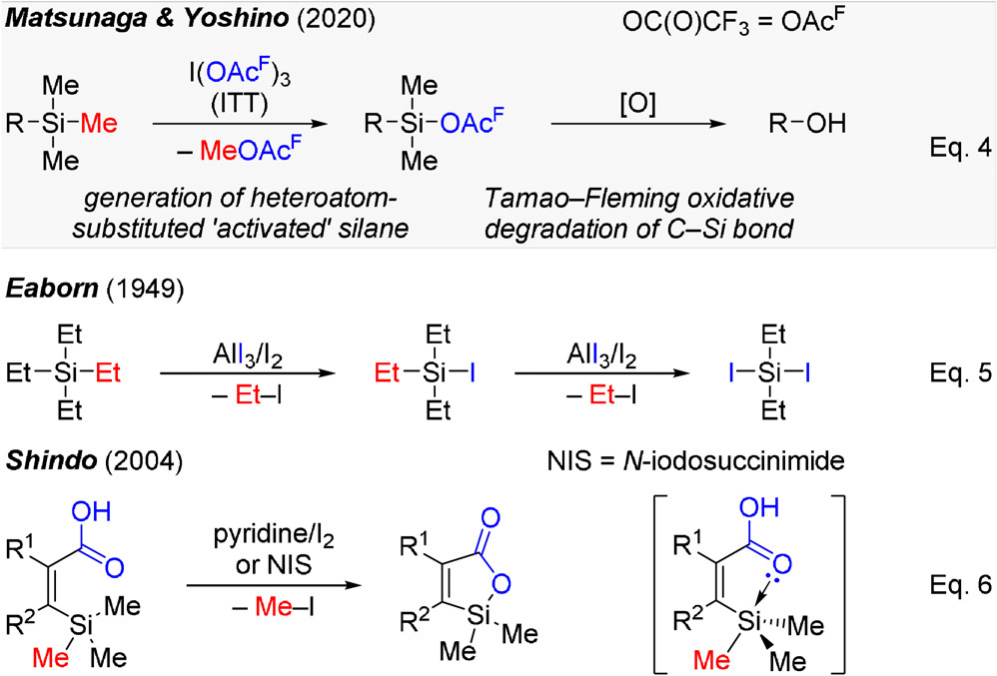



As outlined in Scheme 1 (top), the computed mechanism of the demethylation step reveals that in solution the dimeric ITT complex [ITT]_2_ first converts into more reactive monomeric forms, that is the conformers ITT and ITT′. These then interact with the tetraalkylated silyl group. A concerted methyl group transfer from the silicon atom to the iodine(III) center and formation of a silicon−oxygen bond leads to the generation of a silyl trifluoroacetate derivative (**TS‐I**, bottom). For Me_4_Si, the quantum‐chemical calculations demonstrate an end‐on interaction between the highly electrophilic iodine(III) center and one of the methyl groups. The same mechanistic picture had recently been drawn by Qu and Oestreich for the protolytic demethylation of Me_4_Si.^[10]^ The end‐on interaction of the proton with the back lobe of the σ(C−Si) orbital in **TS‐II** was found to be energetically more favorable than a side‐on interaction with the inner lobe as in **TS‐III** (ΔΔ*G* ^≠^=5.6 kcal mol^−1^, bottom).

**Scheme 1:**
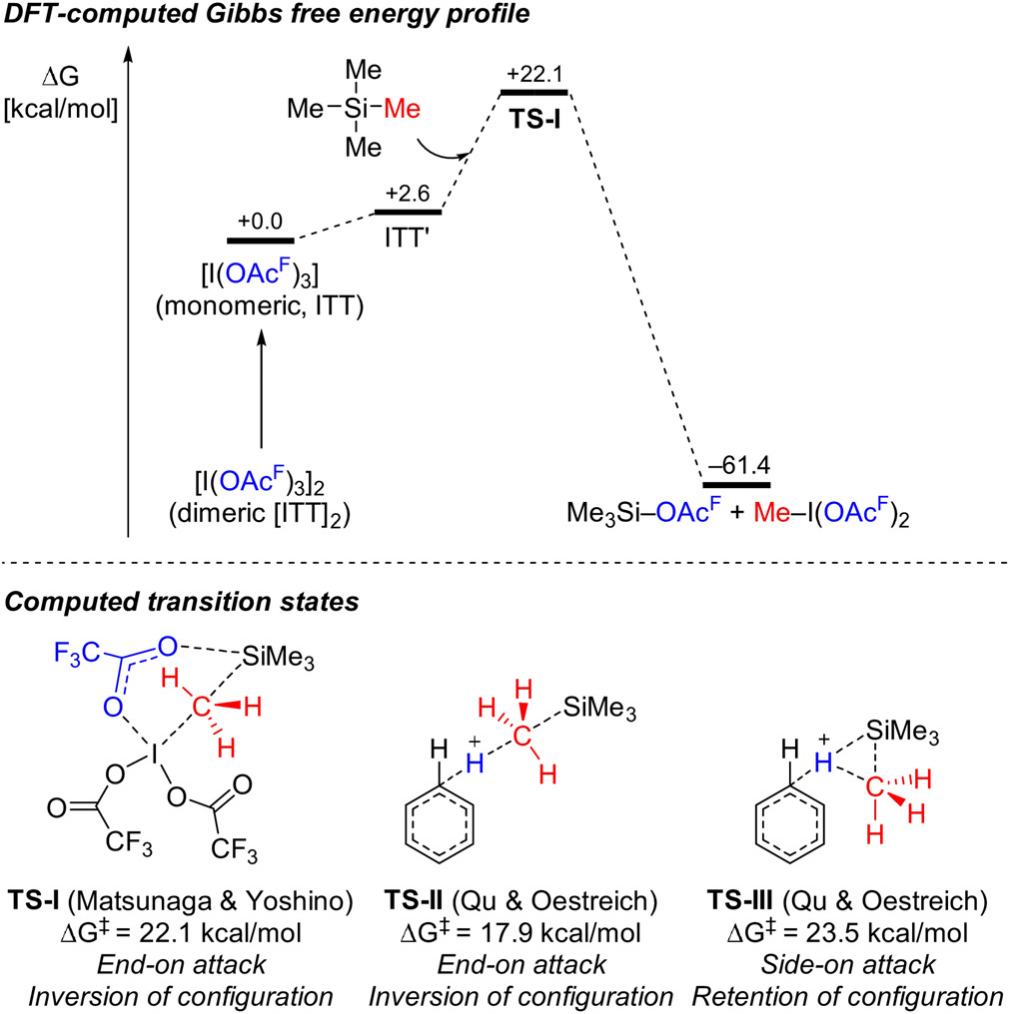
Concerted nucleophile‐assisted electrophilic demethylation of a silicon atom by an electrophilic iodine reagent and, for comparison, protonation of a C(sp^3^)−Si bond.

The methodology introduced by the Matsunaga laboratory shows tremendous functional group tolerance, ranging from any type of carbonyl functionalities to various heterocycles (Scheme 2). Several densely functionalized molecules are compatible with both the demethylation and the oxidative degradation steps. Aside from Me_3_Si, the work also includes examples of Et_3_Si and *t*BuMe_2_Si; their dealkylation proceeds with lower yields.

**Scheme 2:**
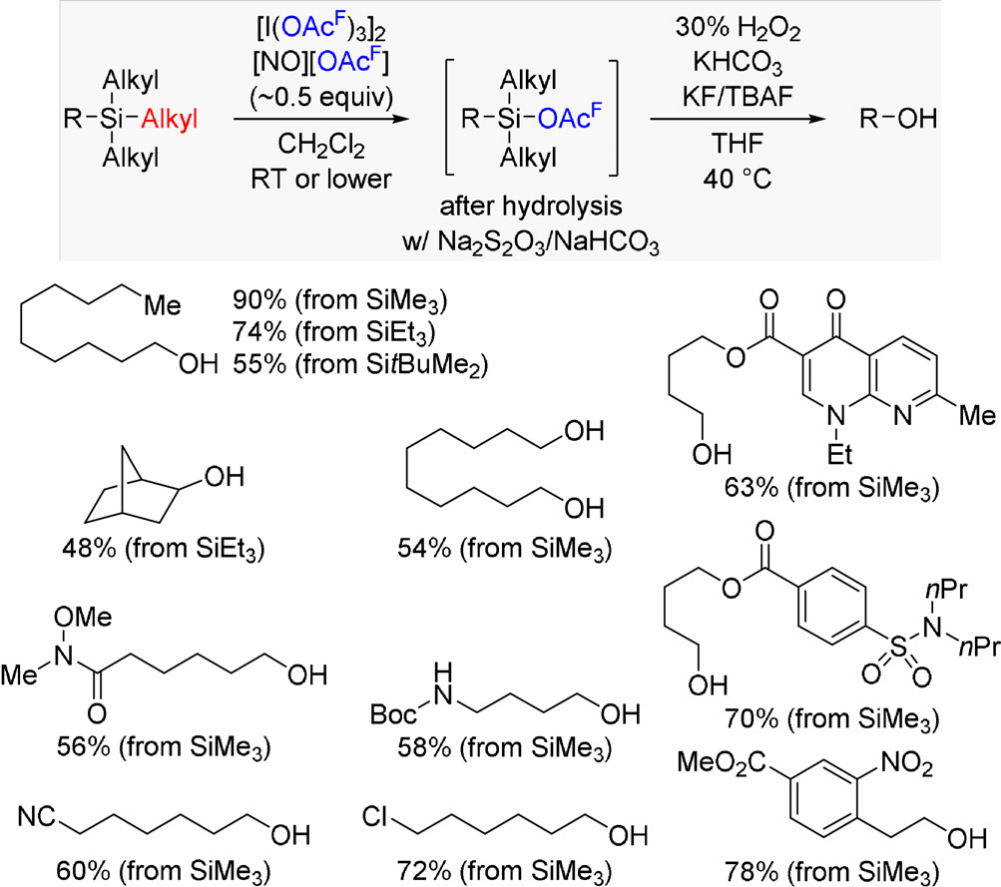
Selected examples of the chemoselective cleavage of a C(sp^3^)−Si bond in unactivated tetraalkylsilanes with ITT followed by oxidative degradation of another C(sp^3^)−Si bond. TBAF=tetra‐*n*‐butylammonium fluoride, Boc=*tert*‐butoxycarbonyl.

Matsunaga, Yoshino, and co‐workers have solved a long‐standing challenge in synthetic methodology, that is, the oxidative degradation of a carbon−silicon bond in unactivated fully alkylated silanes. This brings the “unpopular” Me_3_Si group back to life,^[5]^ thereby significantly expanding the selection of masked hydroxy groups beyond Me_2_PhSi and heteroatom‐substituted Tamao‐type silyl groups. This work is complementary to the traditional protolysis approach but with unprecedented functional group tolerance. Although this methodology is currently limited to tetraalkylsilanes, it will be exciting to see future advances towards the chemoselective cleavage of C(sp^3^)−Si over C(sp^2^)−Si and C(sp)−Si bonds.

## Conflict of interest

The authors declare no conflict of interest.

## References

[1a] F. S. Kipping , L. L. Lloyd , J. Chem. Soc. Trans. 1901, 79, 449–459;

[1b] F. S. Kipping , J. Chem. Soc. Trans. 1907, 91, 209–240.

[2a] C. Eaborn , Pure Appl. Chem. 1969, 19, 375–388 and cited references; for an authoritative review on electrophilic substitution reactions of mainly arylsilanes, see:

[2b] T. H. Chan , I. Fleming , Synthesis 1979, 761–786.

[3a] I. Fleming , R. Henning , H. Plaut , J. Chem. Soc. Chem. Commun. 1984, 29–31; for oxidative degradation of heteroatom-substituted and as such activated silanes, see:

[3b] K. Tamao , M. Akita , M. Kumada , Organometallics 1983, 2, 1694–1698;

[3c] K. Tamao , T. Kakui , M. Akita , T. Iwahara , R. Kanatani , J. Yoshida , M. Kumada , Tetrahedron 1983, 39, 983–990.

[4] G. R. Jones , Y. Landais , Tetrahedron 1996, 52, 7599–7662.

[5] For a multi-step sequence, see: T. Torigoe , T. Ohmura , M. Suginome , J. Org. Chem. 2017, 82, 2943–2956. 28185456 10.1021/acs.joc.6b02917

[6a] L. H. Sommer , R. P. Pioch , N. S. Marans , G. M. Goldberg , J. Rockett , J. Kerlin , J. Am. Chem. Soc. 1953, 75, 2932–2934;

[6b] L. H. Sommer , W. P. Barie , J. R. Gould , J. Am. Chem. Soc. 1953, 75, 3765–3768.

[7a] D. H. O'Brien , C. M. Harbordt , J. Organomet. Chem. 1970, 21, 321–328;

[7b] D. D. Hopf , D. H. O'Brien , J. Organomet. Chem. 1976, 111, 161–169 and cited references.

[8] G. A. Olah , A. Husain , B. G. B. Gupta , G. F. Salem , S. C. Narang , J. Org. Chem. 1981, 46, 5212–5214.

[9] M. Demuth , G. Mikhail , Synthesis 1982, 827.

[10] Q. Wu , Z.-W. Qu , L. Omann , E. Irran , H. F. T. Klare , M. Oestreich , Angew. Chem. Int. Ed. 2018, 57, 9176–9179; 10.1002/anie.201805637 29775241

[11a] C. C. Price , J. R. Sowa , J. Org. Chem. 1967, 32, 4126–4127; see also:

[11b] J. H. Smitrovich , K. A. Woerpel , J. Org. Chem. 1996, 61, 6044–6046.

[12] K. Matsuoka , N. Komami , M. Kojima , T. Mita , K. Suzuki , S. Maeda , T. Yoshino , S. Matsunaga , J. Am. Chem. Soc. 2021, 143, 103–108. 33356223 10.1021/jacs.0c11645

[13] C. Eaborn , J. Chem. Soc. 1949, 2755–2764.

[14] M. Shindo , K. Matsumoto , K. Shishido , Angew. Chem. Int. Ed. 2004, 43, 104–106; 10.1002/anie.200352705 14694484

